# Self-Assembled Membrane-like Nanomaterials from Sequence-Defined Peptoid Block Copolymers

**DOI:** 10.3390/polym13152389

**Published:** 2021-07-21

**Authors:** Tiantian Wei, Jingjing Wu, Xiran Shen, Zhifeng Qiu, Li Guo

**Affiliations:** Research School of Polymeric Materials, School of Materials Science & Engineering, Jiangsu University, Zhenjiang 212013, China; 2211905055@stmail.ujs.edu.cn (T.W.); wujingj0516@163.com (J.W.); 2112005015@stmail.ujs.edu.cn (X.S.); 17315766164@163.com (Z.Q.)

**Keywords:** polypeptoid, self-assembly, membrane-like nanomaterial, block copolymer, sequence-defined polymer

## Abstract

Sequentially defined membrane-like nanomaterials have potential applications in biomedical and chemical fields due to their unique physical and chemical properties. However, these natural and synthetic nanomaterials have not been widely developed due to their complicated molecular sequence and structure, difficulties in synthesis etc. Here, we report a stable membrane-like nanomaterial composed of a monolayer or bilayer that was self-assembled from sequence-defined amphiphilic peptoid triblock (poly(*N*-aminoethyl glycine)-b-poly(*N*-octyl glycine)-b-poly(*N*-carboxyethyl glycine)) and diblock (poly(*N*-carboxyethyl glycine)-b-poly(*N*-octyl glycine) and poly(*N*-aminoethyl glycine)-b-poly(*N*-octyl glycine)) copolymers separately. A series of peptoid block copolymers were synthesized, and it was observed that long alkyl side chains and abundant hydrophobic blocks were necessary to form the membranes. The prepared membrane-like nanomaterials were fairly stable. They did not change obviously in shape and size with time, and they can survive after sonication. This study is expected to enrich the nanomaterial family, as well as polypeptoid science, and expand their applications in biomedicine and other fields.

## 1. Introduction

In the past 20 years, two-dimensional (2D) nanomaterials have attracted an increasing amount of attention because of their unique physical and chemical properties [1,2,3,4,5]. There is a class of 2D nanomaterials assembled from sequence-defined molecules, such as DNA [6,7], proteins [8,9] and peptides [10]. They have excellent properties, unique structural and self-identification characteristics [3,6,7,8,9,10,11,12,13]. For example, cell membranes are composed of the phospholipid bilayer, which is a special 2D nanomaterial with the functions of selective substance transportation, information recognition etc. [14,15]. To mimic the structure, scientists have made many efforts to design, synthesize and construct biomimetic membranes, expecting them to have similar properties [6,14,15,16,17,18]. However, the molecular sequences and structures are complicated, making the synthesis work hard to achieve [7,12]. Moreover, the molecules tend to interact with each other and assemble in unpredictable ways [19,20], and specific raw materials are usually required [21], which greatly limits the development of the material. Therefore, the preparation of sequence-defined membrane-like nanomaterials is still a great challenge.

Polypeptoids (*N*-substituted polyglycines) are a new class of protein mimetic biocompatible materials. They have the same backbone structures as those of polypeptides except that the side chains are transferred from the alpha carbons to the amide nitrogens. Due to the substitution groups moving from C to N, the main chains of polypeptoids are free of hydrogen bonds and chiral centers, which makes the material have special properties that are superior to those of polypeptides [22]. For example, polypeptoids have good solubility, strong resistance to enzymatic hydrolysis, good thermal processing performance and highly controllable chemical structures [23]. As a new biomimetic material to replace polypeptides, polypeptoids have made rapid progress in both synthesis methods and property studies [22,23,24,25,26,27,28,29,30,31,32]. However, only a few groups have explored the construction of membrane-like nanomaterials from polypeptoids. Zuckermann et al. designed and synthesized amphiphilic polypeptoid, which was able to self-assemble into sheet nanomaterial for the first time in 2010 [24]. After that, Chen et al. synthesized diblock polypeptoids with different structures and prepared corresponding membrane-like nanomaterials [13]. These reports revealed the capability of polypeptoids as great candidates to form sequence-defined membrane-like nanomaterials having potential applications in chemistry study and biotechnology, while the structures are very limited and the methods suffer from low efficiency.

Herein, we attempted to construct stable, sequence-defined membrane-like nanomaterials from polypeptoids in a convenient way and point to the design rules of the molecular structures. We designed and prepared a series of sequence-defined amphiphilic peptoid diblock and triblock copolymers with alkyl side chains as the hydrophobic blocks. For the first time, sequence-defined peptoid triblock copolymers were able to self-assemble in an aqueous solution to form a monolayer membrane-like nanomaterial. The prepared peptoid diblock copolymers can form bilayers in water. The effects of lengths of alkyl side chains and hydrophobic blocks on self-assembly behavior were studied to favor the future structure design. In brief, this paper varies the polypeptoid structures and their nanomaterials, which expands the potential applications of polypeptoids in chemistry and biotechnology.

## 2. Materials and Methods

Dichloromethane (DCM), chloroform-d, ether, acetone, methanol, hexane and ethanol were purchased from Sinopharm Chinese Reagent Co (Jiangsu, China). Trifluoroaceticacid, acetonitrile, *n*-octylamine, *n*-butylamine, *n*-hexylamine, dimethyldichlorosilane, *N*-Boc-ethylenediamine β-alanine t-butyl ester hydrochloride, *N*,*N*-diisopropylethylamine, *N*,*N*-dimethylformamide (DMF), triisopropylsilane (TIS), rink amide resin, 4-methylpiperidine, and *N*,*N*′-diisopropylcarbodiimide (DIC) were purchased from Aladdin. All solvents and reagents were used without further purification.

Into rink amide resin (100 mg), 2 mL DMF was added and stirred with nitrogen bubbling for 10 min. DMF was drained by vacuum to separate swelled resin. 1 mL of 20% 4-methylpiperidine in DMF (*v*/*v*) solution was added to deprotect the Fmoc group, stirred for 2 min, and then drained. The above step was repeated, but the stirring time was changed to 12 min. Then, 2 mL DMF was added to rinse the resin for 15 s and drained. This was repeated 4 times. The amine-functionalized resin was bromoacylated for 20 min by adding bromoacetic acid (0.5 mL, 1 M in DMF) and DIC (0.5 mL, 1 M in DMF), and then rinsed with DMF 5 times. An amount of 1 mL of primary amine DMF solution (2 M) was added and stirred. After 30 min, the resin was rinsed with DMF 5 times. The designed peptoid copolymers were synthesized by repeating the above two steps of bromoacylation and amine displacement. After the final step, the resin was washed with DMF (5 × 15 s) and DCM (3 × 15 s), dried and stored at −18 °C.

Peptoid block copolymers were cleaved with 4 mL of trifluoroacetic acid (TFA) cleavage cocktail (TFA/H_2_O/TIS = 95/2.5/2.5, *v*/*v*/*v*) for 2 h followed by blowing a stream of N_2_ gas to evaporate the cocktail. The oily products obtained were purified by preparative RP-HPLC and lyophilization. The pure products were identified by MS analysis. The purity of each final product was confirmed by analytical RP-HPLC using an analytical column (SHIMADZU Inertsil OSD-SP, 4.6 mm × 250 mm, 5 μm).

The purified sample was dissolved into a mixed solvent of water/acetonitrile (*v*/*v* = 1) at a concentration of 5 mM. Then, 0.5 mL of the solution was slowly volatilized in the hood at room temperature until it became turbid or gelatinous to form a self-assembled structure.

A small amount of self-assembled sample was diluted with pure water and then dropped onto a silicon wafer. After the liquid on the silicon wafer was dried, the sample was characterized using a field emission scanning electron microscope (FESEM, FEI NovaNano 450, Hillsboro, OR, USA) or an atomic force microscope (AFM, MFP-3D, Mankato, MN, USA) to observe the morphology of the self-assembled sample.

The self-assembled samples in aqueous solutions were placed into a bath sonicator (Kunshan Shumei KQ3200E, Suzhou, China, 40 KHz,150 W) and exposed to sonication. After 15 and 30 min, the samples were characterized by SEM to check the stability of the self-assembled structures.

## 3. Results and Discussion

### 3.1. Design and Synthesis of Polypeptoids

**Figure 1:**
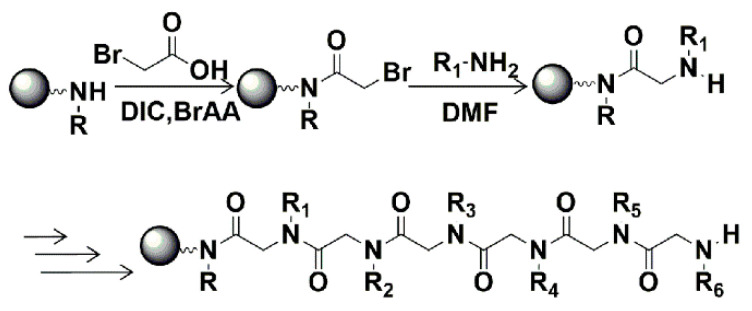
Schematic diagram of peptoid solid-phase submonomer synthesis method.

**Figure 2:**
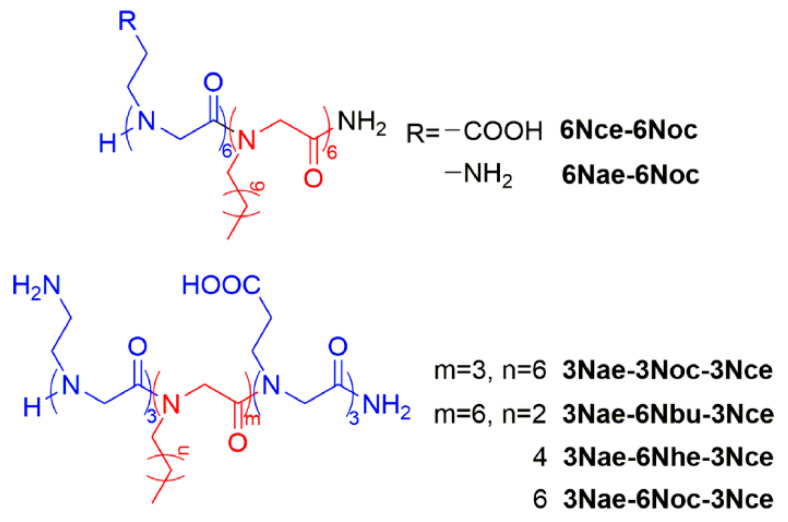
Structures of sequence-defined peptoid block copolymers.

Sequence-defined polypeptoids can be easily obtained via the solid-phase submonomer synthesis method (Figure 1), which was developed by Zuckermann et al. [33]. It gives an efficient way to prepare polypeptoids with varying structures. With this method, we designed and synthesized a series of amphiphilic peptoid block copolymers (Figure 2). Different side chains were introduced by primary amine submonomers in the displacement steps. The residues with octyl, hexyl and butyl side chains formed the hydrophobic blocks of the polypeptoid molecules, and the hydrophilic blocks contained the residues with carboxyethyl or aminoethyl side chains. Here, we used long alkyl chains as hydrophobic groups, instead of aromatic substitutes reported in previous studies, to enhance the submonomer reaction activities and decrease the toxicity. Polypeptoids with long alkyl side chains have good crystallinity [22], which can serve as the driving force for self-assembly. For triblock copolymers, the hydrophilicity comes from two blocks separately at the polymer chain ends with carboxyethyl or aminoethyl side chains. The purpose of this design is to stabilize the assembled nanomaterial with extra Coulomb force between carboxyl and amino groups. Alkyl side chains with different lengths (octyl, hexyl and butyl) and varying hydrophobic block lengths (three residues and six residues) were designed to compare their effects on self-assembling. All synthesized peptoid copolymers were purified with reverse-phase high-performance liquid chromatography (RP-HPLC), and the structures and purities were confirmed by mass spectrometry and HPLC separately (Figures S2–S13).

### 3.2. Self-Assembly of Peptoid Block Copolymers into Membrane-Like Nanomaterials

**Figure 3:**
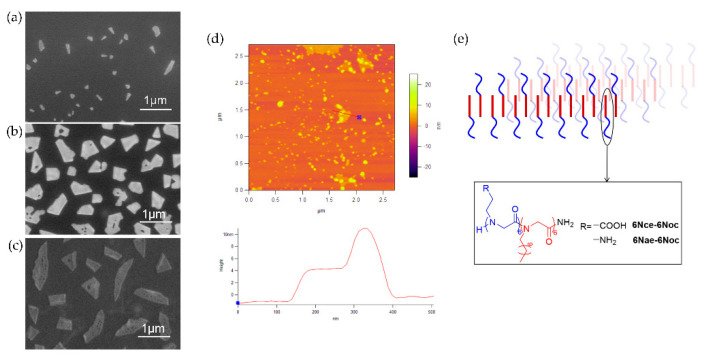
SEM images of self-assembled membrane-like nanomaterials from (**a**) 6Nce-6Noc, (**b**) 6Nae-6Noc and (**c**) the mixture of 6Nce-6Noc/6Nae-6Noc (1/1); (**d**) AFM image of self-assembled membrane-like nanomaterials from 6Nae-6Noc and the cursor profile for the line in the image; (**e**) illustration of bilayer formed from peptoid diblock copolymers.

**Figure 4:**
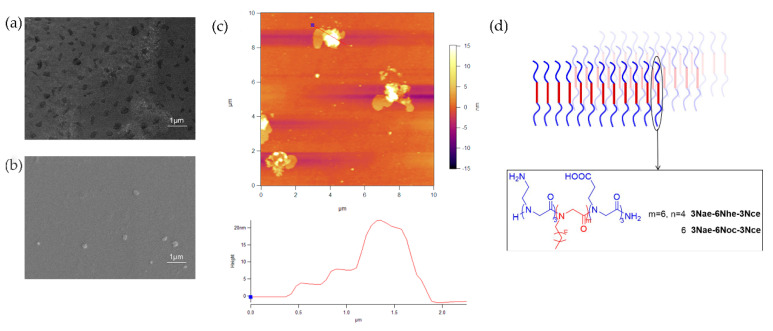
SEM images of self-assembled membrane-like nanomaterials from (**a**) 3Nae-6Noc-3Nce and (**b**) 3Nae-6Nhe-3Nce; (**c**) AFM image of self-assembled membrane-like nanomaterials from 3Nae-6Noc-3Nce and the cursor profile for the line in the image; (**d**) illustration of monolayer formed from peptoid triblock copolymers.

The prepared amphiphilic peptoid block copolymers were dissolved in a mixed solvent of water/acetonitrile. With the evaporation of acetonitrile, water turned into the main solvent. The hydrophobic blocks, free extended in the mixed solvent, became insoluble in water. The intrinsic hydrophobicity of blocks having crystalline alkyl side chains drove the polypeptoid molecules to assemble into membrane-like nanomaterials (Figure 3 and Figure 4). For peptoid diblock copolymers, six residues with octyl side chains composed hydrophobic blocks, and six residues with carboxyethyl or aminoethyl side chains made the hydrophilic blocks. SEM and AFM results (Figure 3a,b,d) indicate that both diblock copolymers can form irregular shaped membrane-like nanomaterials, while the sizes are different. The membranes assembled from 6Nae-6Noc are about 500 nm in size, much larger than that from 6Nce-6Noc (about 100 nm). To further stabilize the membrane, 6Nae-6Noc and 6Nce-6Noc were dissolved in water/acetonitrile together with a ratio of 1:1, so the interactions between carboxyl and amino groups could favor the assembly. The results suggest that mixing two polypeptoids together did help them to form larger membranes, from 100 nm or 500 nm to 800 nm (Figure 3c). AFM analysis of the membranes from diblock copolymers suggests that the membrane thickness is around 5 nm (Figure 3d), which confirms the formation of bilayers as expected (Figure 3e). It is also similar to the thickness of the lipid bilayer membrane [14,15]. There are a lot of overlapped membranes observed in AFM that could be formed by stacking during sample preparation. Some small structures in AFM, which are invisible in SEM due to their small size (about 10 nm), may be the membranes at the initial growth stage. The nanomaterials formed here are very similar to the membrane or sheet structures prepared in previous studies [6,13,24], although the sizes are smaller. This is because the hydrophobic interactions of alkyl side chains are weaker than that of aromatic substitutes.

The peptoid triblock copolymers we designed have hydrophobic blocks in the middle of the polymer chains and hydrophilic blocks at both chain ends (3Nae-6Noc-3Nce). We kept the hydrophobic blocks with residues having octyl side chains since they had enough hydrophobicity to induce assembly, as shown for diblock copolymers. The two hydrophilic blocks at both chain ends have carboxyl and amino groups separately, which can further stabilize the assembled structures. As expected, the molecules self-assembled into membrane-like nanomaterials (Figure 4), which gave the first example of membranes from sequence-defined peptoid triblock copolymers. Compared with the membranes from diblock copolymers, membranes from triblock copolymers have similar shapes but smaller sizes, about 200 nm (Figure 4a). AFM analysis (Figure 4c) revealed that the peptoid triblock copolymers form monolayer membranes with a thickness of 4 nm, as shown in Figure 4d.

### 3.3. Effects of Hydrophobic Structures on Self-Assembly

To study the polypeptoid structure effects, especially the hydrophobic block structure effects, on self-assembly behavior, peptoid triblock copolymers with different lengths of alkyl side chains and hydrophobic blocks were designed and synthesized. In the solid-phase submonomer synthesis method, shortening the polymer chain length will reduce synthetic time and increase efficiency, which can greatly decrease synthesis work. We reduced the hydrophobic blocks from six residues (3Nae-6Noc-3Nce) to three (3Nae-3Noc-3Nce), and no membranes were observed, which suggests that six residues of hydrophobic blocks are necessary for membrane formation. In the further study, we kept the hydrophobic block lengths but shortened the alkyl side chains, from eight carbons (3Nae-6Noc-3Nce) to six (3Nae-6Nhe-3Nce) and four carbons (3Nae-6Nbu-3Nce). It is clearly shown by SEM characterization that no membranes formed from 3Nae-6Nbu-3Nce, suggesting the lack of hydrophobic interactions. Replacing butyl with hexyl, 3Nae-6Nhe-3Nce had sufficient hydrophobic interactions to assemble into membranes (Figure 4b), although the membrane size was smaller than that of 3Nae-6Noc-3Nce. The results indicate that enough hydrophobicity is critical for nanomaterial formation. According to previous studies, six hydrophobic residues with hexyl side chains did not have enough driving force to assemble peptoid diblock copolymers into membranes [6], which is different from our results. This demonstrates the advantage of our triblock copolymer design. Besides the hydrophobic interactions of hexyl groups, the interactions between carboxyl and amino groups also contribute to facilitating molecule assembly. 

### 3.4. Stability of the Membrane-like Nanomaterials

**Figure 5:**
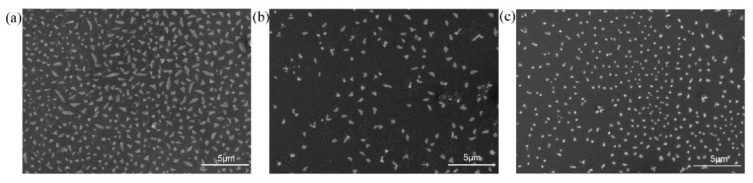
SEM images of the membrane-like nanomaterials from the mixture of 6Nce-6Noc/6Nae-6Noc (1/1) (**a**) before, (**b**) 15 min after and (**c**) 30 min after sonication.

The assembled membrane-like nanomaterials should be quite stable in solvents and at high temperatures, as reported by Chen et al. [6]. Here, we further studied their stability. The prepared membranes were kept in the aqueous solution of free polypeptoid molecules. The size and shape of the membranes did not have obvious change after 2 days and 6 days (Figure S1), which suggests the membranes are stable over time. To test their resistance to mechanical stress, the polypeptoid membranes were exposed to sonication in a bath sonicator for 15 and 30 min. The SEM images indicate that the membranes survived after sonication, although the size shrank (Figure 5). Therefore, the nanomaterials we prepared here have relatively good mechanical stability.

## 4. Conclusions

In summary, we designed and synthesized a series of sequence-defined amphiphilic peptoid block copolymers that were able to self-assemble into membrane-like nanomaterials. For the first time, membranes from peptoid triblock copolymers were prepared. The hydrophobic interactions of alkyl side chains play a key role to form the membranes. Therefore, enough alkyl chain length and hydrophobic block length are necessary for the self-assembly. The interactions between carboxyl and amino groups in hydrophilic block side chains can help stabilize the assembled nanomaterials. The prepared membranes are fairly stable over time and can survive after sonication. This study provides a good template to synthesize and prepare membrane-like nanomaterials, enriches the structures and broadens their applications in chemistry and biotechnology.

## Supplementary Materials

The following are available online at https://www.mdpi.com/article/10.3390/polym13152389/s1, Figure S1: SEM images of the membrane-like nanomaterials from 6Nae-6Noc after 2 days and 6 days., Figure S2–S7: HPLC traces of purified peptoid block copolymers. Figure S8–S13: Mass-spectrometry analysis for peptoid block copolymers. Figure S14 and S15: ^1^H NMR spectra of peptoid block copolymers.
