# Supplementary material for: Self-Assembled Membrane-like Nanomaterials from Sequence-Defined Peptoid Block Copolymers
Source: Polymers (Basel). 2021 Jul 21;13(15):2389. doi: 10.3390/polym13152389 (PMC8346955; doi:10.3390/polym13152389)
Supplement: Supplementary file 1 [file polymers-13-02389-s001.zip › polymers-1300909-supplementary.pdf]

polymers

Supplementary Material

Self-assembled Membrane-like Nanomaterials from Sequence-defined Peptoid Block Copolymers

Tiantian Wei[1,†], Jingjing Wu[1,†], Xiran Shen[1], Zhifeng Qiu[1], Li Guo[1*]

[1] Research School of Polymeric Materials, School of Materials Science & Engineering, Jiangsu University, Zhenjiang, 212013, China
* Correspondence: liguo@ujs.edu.cn
† These authors contributed equally to this work.

Supplementary Figures

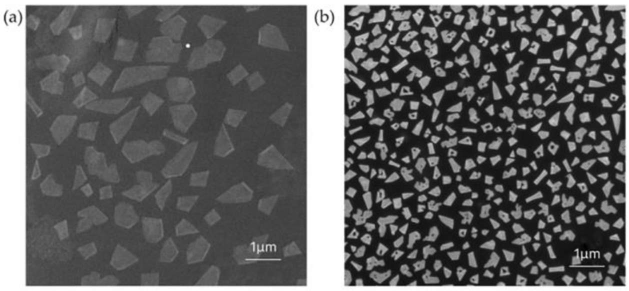

Figure S1. SEM images of the membrane-like nanomaterials from 6Nae-6Noc after (a) 2 days and (b) 6 days.

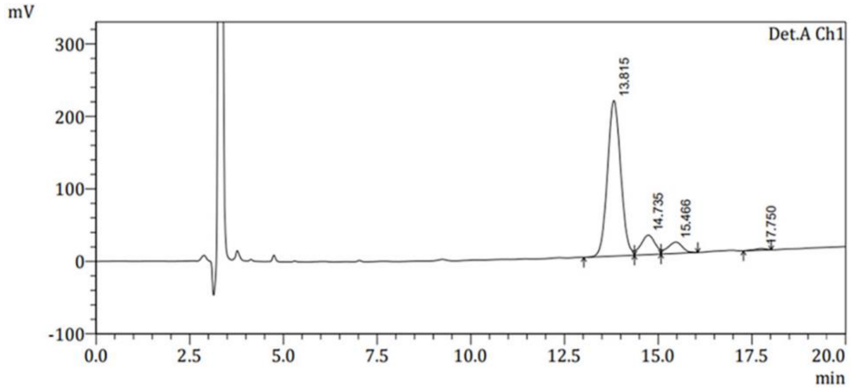

Figure S2. HPLC trace of purified 6Nce-6Noc.

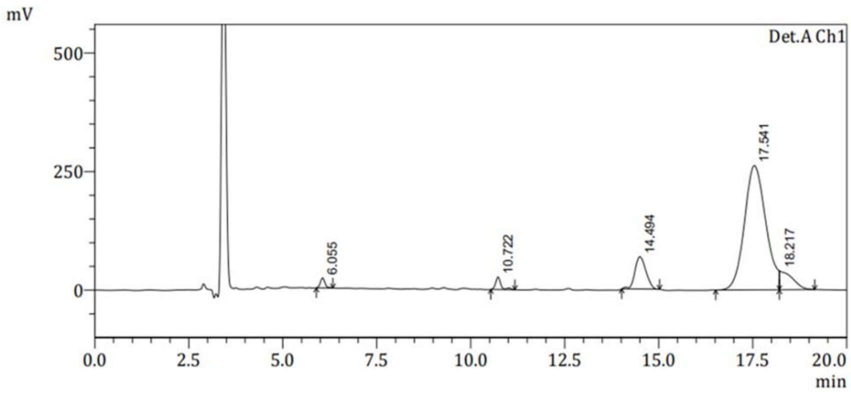

Figure S3. HPLC trace of purified 6Nae-6Noc**.**

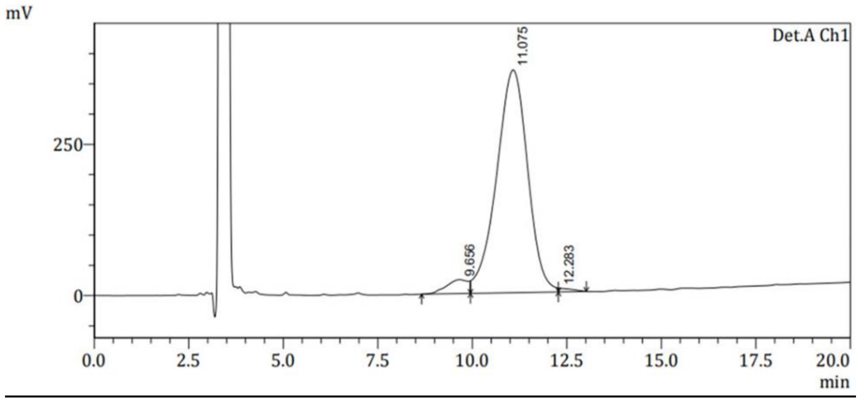

Figure S4. HPLC trace of purified 3Nae-6Noc-3Nce.

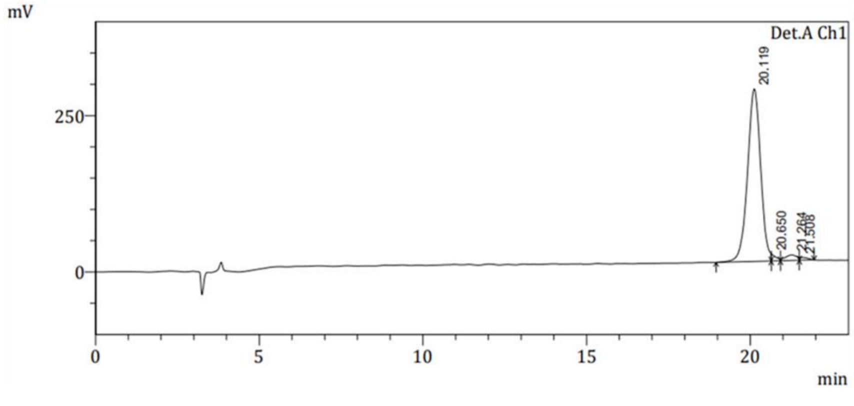

Figure S5. HPLC trace of purified 3Nae-6Nhe-3Nce.

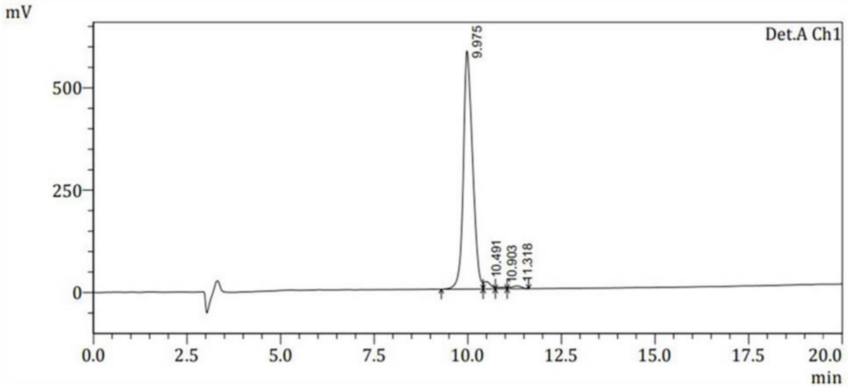

Figure S6. HPLC trace of purified 3Nae-3Noc-3Nce.

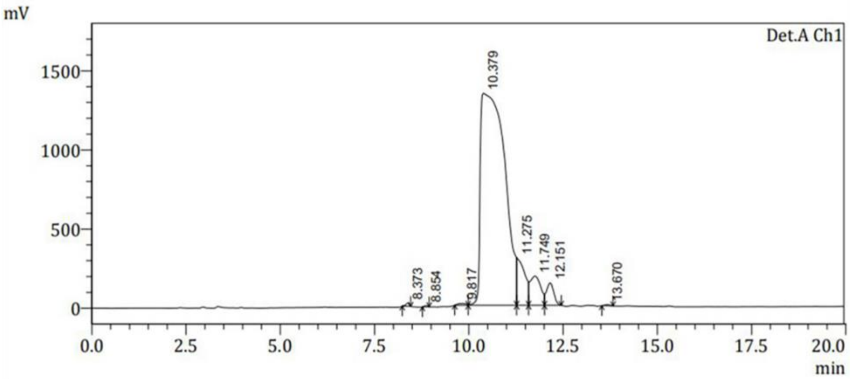

Figure S7. HPLC trace of purified 3Nae-6Nbu-3Nce.

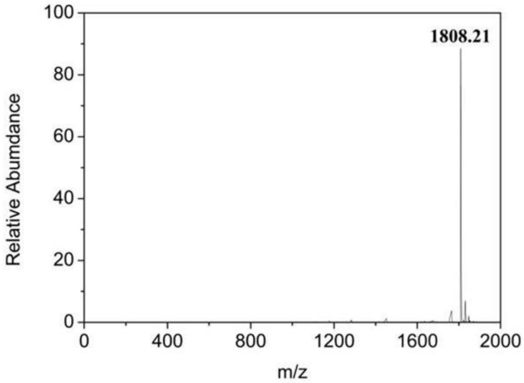

Figure S8. Mass-spectrometry analysis for 6Nce-6Noc.

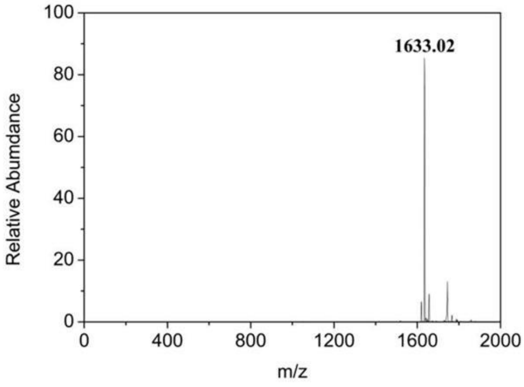

Figure S9. Mass-spectrometry analysis for 6Nae-6Noc.

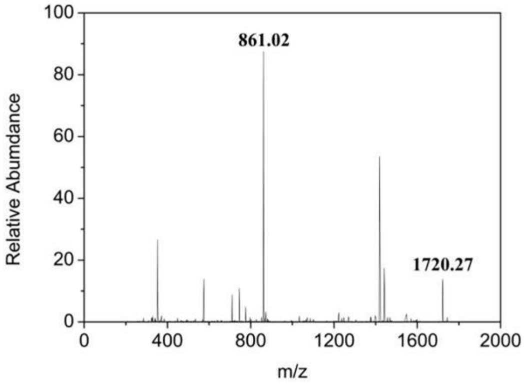

Figure S10. Mass-spectrometry analysis for 3Nae-6Noc-3Nce.

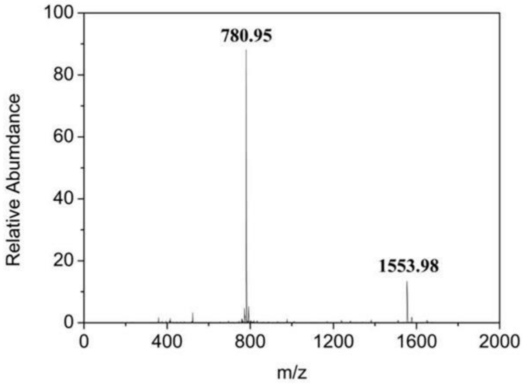

Figure S11. Mass-spectrometry analysis for 3Nae-6Nhe-3Nce.

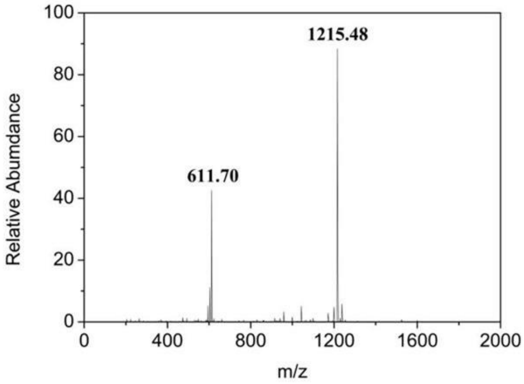

Figure S12. Mass-spectrometry analysis for 3Nae-3Noc-3Nce.

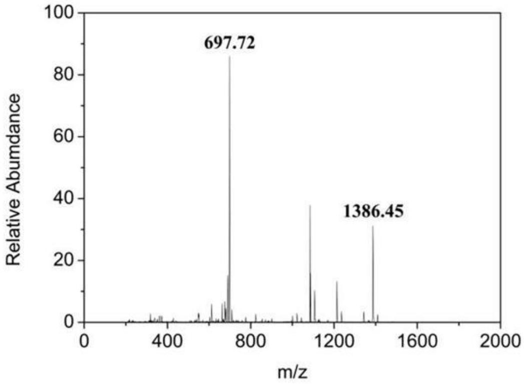

Figure S13. Mass-spectrometry analysis for 3Nae-6Nbu-3Nce.
